# Opiate use inhibits TLR9 signaling pathway *in vivo*: possible role in pathogenesis of HIV-1 infection

**DOI:** 10.1038/s41598-017-12066-3

**Published:** 2017-10-12

**Authors:** Yanyan Liao, Junjun Jiang, Bingyu Liang, Fumei Wei, Jiegang Huang, Peijiang Pan, Jinming Su, Bo Zhou, Ning Zang, Li Ye, Hao Liang

**Affiliations:** 1 Guangxi Key Laboratory of AIDS Prevention and Treatment & Guangxi Universities Key Laboratory of Prevention and Control of Highly Prevalent Disease, School of Public Health, Guangxi Medical University, Nanning, China; 2 Guangxi Collaborative Innovation Center for Biomedicine, Life Science Institute, Guangxi Medical University, Nanning, China

## Abstract

The molecular mechanism of opiate use promoting HIV-1 infection is not fully understood. TLR9 is expressed in many immune cells, including monocytes, macrophages, which can recognize viruses and viral products and consequently induce the production of antiviral factors and initiate immune responses. Previous studies have shown that chronic viral infections can overcome and impair TLR9 pathway. We aimed to explore whether opiate use enhances HIV infection through inhibition of TLR9 pathway via a population-based study. A total of 200 subjects were enrolled and divided into four groups as follows: Opiate+ HIV+ (50), Opiate− HIV+ (50), Opiate+ HIV− (50), and healthy control (Opiate− HIV−, 50). All HIV-infected subjects did not receive antiretroviral therapy while they were enrolled in the study. The results showed that opiate use was associated with higher viral load and lower CD4+ T cell count. Opiate use alone led to lower expression of TLR9, IRF7, and IFN-α at the protein level in PBMCs. Combined with HIV-1 infection, opiate use resulted in lower expression of MyD88, ISG56, and MxA. In addition, morphine treatment promoted HIV-1 replication in macrophages via inhibition of TLR9 pathway. Our data reveal that opiate use plays a cofactor role in pathogenesis of HIV-1 infection through inhibition of TLR9 pathway.

## Introduction

Opiates are a kind of powerful and highly addictive anesthetic, consisting of heroin, morphine, opium and cocaine. Opiate users are at a high risk for human immunodeficiency virus-1 (HIV-1) infection due to high-risk behaviors, such as sharing syringes, unprotected sex and pharmacological drug effects. The prevalence of HIV-1 infection among people who inject drugs is approximately 28% in Asia^1^. There were approximately 2.098 million drug addicts in China by the end of 2012, and among them, 1.272 million were opiate users, accounting for as high as 60.6% of the total addicts^2^.

Opiate use can promote HIV-1 infection, increase viral load, and accelerate the process of acquired immune deficiency syndrome (AIDS), which has been confirmed in a clinical trial^3^, an epidemiological investigation^4^, and experiments *in vivo and in vitro* ^5,6^. Over 30 months, the viral load is significantly higher in crack-cocaine users independent of highly active antiretroviral therapy (HAART) compared to non-users^7^. In addition, chronic opiate use compromises the immune system^8–10^ and thereby increases the risk of HIV infection^11–14^. In addition, morphine has the ability to enhance HIV-1 replication in MT2 cells or macrophages and to reduce the lamivudine antiviral effect in the early stage of infection *in vitro* ^15,16^. However, there is still a discrepancy that fentanyl (morphine derivative) has no effect on HIV production from peripheral blood cells^17^. One possible reason for this discrepancy is that the effects of the morphine derivative on HIV production may be different than those from morphine. Importantly, several other studies have confirmed that morphine enhances HIV infection in several peripheral blood mononuclear cells (PBMCs), such as macrophages and CD4^+^ T cells^18,19^. Therefore, the molecular mechanism of opiate use in the pathogenesis of HIV-1 infection is not fully understood.

The innate immune system is the body’s first line of defense against pathogen infections and an important barrier against infectious diseases. The hallmark of innate immunity is its rapid ability to recognize a series of pathogen-associated molecular patterns (PAMPs) via toll-like receptors (TLRs) and other immune sensors^20,21^. TLR9 is a member of the Toll-like family of receptors^22,23^, which is mainly expressed on monocytes, macrophages, dendritic cells, B cells and T cells^24^. It can detect the molecular structures of invading microbial pathogens to sense infection and then initiate innate immune responses^25^. In addition, several reports have confirmed that chronic viral infections can impair TLR9 signaling and down-regulate TLR9 gene expression^26–29^. TLR9 can recognize cytosine phosphate guanosine (CpG) motifs in viral DNA and then activate interferon regulatory factor 7 (IRF7), which leads to type I IFN secretion by plasmacytoid dendritic cells (pDCs)^30,31^. HIV, HBV, HCV, Epstein Barr virus (EBV) and Human Papillomavirus (HPV) can interact with regulatory receptors on pDCs to impair TLR9 signaling and down regulate TLR9 gene expression^32,33^. Recently, the role of TLR9 in restricting virus replication and modulating innate immunity has been discovered^26,34–36^. However, there is little information about whether opiate use has an impact on the TLR9 signal pathway in the context of HIV infection.

A population-based study previously conducted by our group^37^ showed that opiate use is associated with lower expression of TLR9 mRNA during HIV infection. In the present study, we continued to examine the expression of TLR9 protein and its downstream factors and explored whether opiate use inhibits the TLR9 signaling pathway and thereby modulates innate immune function, thus facilitating the pathogenesis of HIV-1 infection.

## Materials and Methods

### Samples

A total of 200 subjects were recruited from methadone maintenance treatment clinics or HIV voluntary counseling and testing clinics in the cities of Nanning, Liuzhou, and Qinzhou in Guangxi, China. Among them, 50 subjects were opiate-abuse and HIV-infected subjects (Opiate+ HIV+), 50 were non-opiate-abuse and HIV-infected (Opiate− HIV+), 50 were opiate-abuse and HIV-uninfected (Opiate+ HIV−), and 50 were healthy subjects (Opiate− HIV−, control). The HIV+ subjects in this study were all in the chronic stage of HIV infection. Blood samples of all participants were screened by enzyme-linked immunosorbent assay (ELISA) and western blot (WB) to confirm their sero-positive or sero-negative status for HIV-1. Opiate use was assessed by self-report and confirmed by a urine test. The individuals recruited in this population-based study met the following eligibility criteria: 1) aged 18 to 60 years old; 2) the HIV-1 infected subjects were ART-naïve, had no HIV symptoms, and their CD4^+^ T cells > 400 cells/μL; and 3) the opiate abusers had taken/injected opiate drugs at least once per year. We excluded substance abusers who had used other drugs besides opiates, such as methamphetamine and ketamine, in the previous six months, had received anti-retroviral therapy, had any other chronic diseases, or could not answer the questionnaires independently.

### Ethics Statement

Written informed consent was obtained from each participant prior to their enrollment. All methods were carried out in accordance with relevant guidelines and regulations, and all experimental protocols were approved by the Ethics and Human Subjects Committee of Guangxi Medical University.

### Specimen collection, PBMC isolation, Generation of monocyte-derived macrophages, and Cell culture

For each participant, PBMCs were isolated from 15 mL heparin-anticoagulated blood using the standard Ficoll-Hypaque centrifugation procedure. Cell counts and cell viability were determined by trypan blue dye. In addition, 5 mL of EDTA-anticoagulated blood was obtained from each participant to quantify HIV-1 viral load and CD4+ T-cell count.

### Plasma viral load, CD4+ T cell count, Monocyte detection

HIV-1 viral load was determined by the reverse transcriptase-polymerase reaction using the Roche Amplicor reagents (Branchburg, NJ) following the manufacturer’s instructions. CD4^+^ T-cell count was measured by flow cytometry using TriTESTCD4FITC/CD8PE/CD3PerCP reagent from Becton, Dickinson and Company (Franklin, NJ). Monocytes in the blood samples of healthy controls (Opiate− HIV−) were displayed by flow cytometry using PerCP-Cy™5.5 Mouse Anti-Human CD14, and the level of TLR9 expression in monocytes was displayed using APC Rat anti-Human TLR9. All antibodies for flow cytometry were purchased from BD Biosciences (San Jose, CA, USA). PBMCs were fixed and permeabilized using BD Cytofix/Cytoperm™ reagents, followed by staining with PerCP-Cy™5.5 Mouse Anti-Human CD14 and APC Rat anti-Human TLR9 following protocols by BD Biosciences. Flow cytometric data were acquired using a BD FACSCanto II flow cytometer and analyzed with BD FACSDiva software (BD Biosciences).

### Quantitative real-time PCR (qRT-PCR)

Total cellular RNA was isolated from PBMCs using TriReagent (Molecular Research Center, Cincinnati, OH) as previously described^38^. Total cellular RNA (1 μg) was then subjected to reverse transcription using reverse transcriptase (Promega, Madison, WI). qRT-PCR was performed to detect the mRNA expression of TLR9 signaling pathway-related genes, such asTLR9, myeloid differentiation primary 88 (MyD88), IFN-α, IRF7, interferon-stimulated gene 56 (ISG56) and myxovirus resistance protein A (MxA) and house-keeping gene glyceraldehyde-3-phosphate dehydrogenase (GAPDH), with the iQ SYBR Green Supermix (Bio-Rad Laboratories, Hercules, CA) as previously described^38^. The data were analyzed using the MyiQ software provided by the thermocycler (iCycler iQ real time PCR detection system; Bio-Rad Laboratories). The level of GAPDH mRNA was used as an endogenous reference to normalize the expression of target genes. The special oligonucleotide primers used in this study were synthesized by Beijing Genomics Institute (Shenzhen, Guangdong), and the sequences are listed in Table 1.

**Table 1:** Primer Pairs for Real-Time RT-PCR in this study.

Primer	Orientation	Sequences
GAPDH	Sense:	5′-GGTGGTCTCCTCTGACTTCAACA-3′
	Antisense:	5′-GTTGCTGTAGCCAAATTCGTTGT-3′
TLR9	Sense:	5′-TACCAACATCCTGATGCTAGACTC-3′
	Antisense:	5′-TAGGACAACAGCAGATACTCCAGG-3′
MyD88	Sense:	5′-TGGGTCCTTTCCAGAGTTTG-3′
	Antisense:	5′-GCACATGGGCACATACAGAC-3′
IRF-7	Sense:	5′-TGGTCCTGGTGAAGCTGGAA-3′
	Antisense:	5′-GATGTCGTCATAGAGGCTGTTGG-3′
IFN-α	Sense:	5′-TTTCTCCTGCCTGAAGGACAG-3′
	Antisense:	5′-GCTCATGATTTCTGCTCTGACA-3′
ISG56	Sense:	5′-GCTGAAGTGTGGAGGAAAGAAT-3′
	Antisense:	5′-CTTAGGGGAAGCAAAGAAAATG-3′
MxA	Sense:	5′-TCTGTAAATCTCTGCCCCTGTT-3′
	Antisense:	5′-TCGTGTCGGAGTCTGGTAAAC-3′

### Western blot

Total cell lysates from PBMCs were prepared using a radio immune precipitation assay (RIPA) buffer (Promega, Madison, WI) with 1% protease inhibitor cocktail (Sigma-Aldrich, St. Louis, MO). Protein concentrations were determined by DC protein assay kit (Bio-Rad, Hercules, CA). Equal amounts of cell lysates (20 μg) were separated on 8% to 10% sodium dodecyl sulfate polyacrylamide gel electrophoresis (SDS PAGE) precast gels and transferred to a polyvinylidene fluoride membrane (Millipore, Eschborn, Germany). Membranes were washed with Tris-buffered saline-Tween 20 (TBST), blocked in TBST containing 5% nonfat milk at room temperature for 1 hour, and then incubated overnight at 4 °C with following primary antibodies: mouse anti-β-act in antibodies (1:5000), mouse anti-TLR9 antibody (1:500), mouse anti-MyD88 (1:2000), rabbit anti-IRF7 antibody (1:1000), mouse anti-ISG56 antibody (1:500), and rabbit anti-MxA antibody (1:500). All these antibodies were purchased from Abcam, Inc. (Cambridge, MA). The membranes were then incubated for 1 hour at room temperature with horseradish peroxidase-conjugated anti-mouse IgG or anti-rabbit IgG (1:5000, Abcam Inc., Cambridge, MA). Blots were developed with Super Signal West Pico Chemiluminescent Substrate (Thermo Fisher Scientific, Waltham, MA). Densitometric analysis was performed using ImageJ 1.44 software (National Institutes of Health, USA).

### ELISA

The protein level of IFN-α in plasma was measured by cytokine-specific ELISA kit (e Bioscience, San Diego, CA). Assays were performed according to the manufacturer’s instructions.

### Detection of the effects of morphine on HIV-1 replication/TLR9 expression in macrophages *in vitro*

To further detect whether opiate use plays a role in pathogenesis of HIV-1 infection, seven-day-cultured macrophages derived from monocytes were incubated with or without 10^−6^ mol/L morphine for 24 hours before infection of HIV Bal strain for 2 hours and then cultured for 12 days. The levels of HIV RNA in cultured supernatants were determined by qRT-PCR at 4, 6, 8, 10 and 12 days postinfection. In addition, seven-day-cultured macrophages were incubated with or without different concentrations (10^−12^, 10^−10^, 10^−8^, and 10^−6^ mol/L) of morphine for 24 hours and then incubated with HIV Bal strain for 2 hours in the presence or absence of morphine. After 8 days postinfection, the levels of HIV RNA were determined. Moreover, seven-day-cultured macrophages were incubated with or without different concentrations (10^−12^, 10^−10^, 10^−8^, and 10^−6^ mol/L) of morphine for 24 hours and then the levels of TLR9 mRNA were determined. In addition, ODN2216, the activator of the TLR9 signaling pathway, and its control, ODN2243, were used to treat macrophages to investigate whether the TLR9 signaling pathway plays a role in preventing morphine-enhanced HIV-1 replication. In brief, seven-day-cultured macrophages were pretreated with ODN2216 or ODN2243, then incubated with or without 10^−6^ mol/L morphine for 24 hours before infection of HIV Bal strain for 2 hours, and then cultured for 12 days. The levels of HIV RNA in cultured supernatants were determined at 8 days postinfection.

### Data analysis

The differences between different groups were analyzed using chi-square for categorical variables, analysis of variance for normally distributed variables, and Kruskal-Wallis and Mann-Whitney analyses for non-normally distributed variables. The data, where appropriate, were expressed as the mean ± standard deviation. The correlation between viral load and CD4^+^ T cell count was evaluated using Pearson’s correlation coefficient. Statistical significance was defined as a probability of *P* < 0.05. Statistical analyses were performed with SPSS16.0 (SPSS Inc., Chicago, Illinois, USA), and graphs were created using GraphPad Prism, version 5.0 (GraphPad Software Inc., San Diego, California, USA).

## Results

### Study population and baseline characteristics

**Figure 1:**
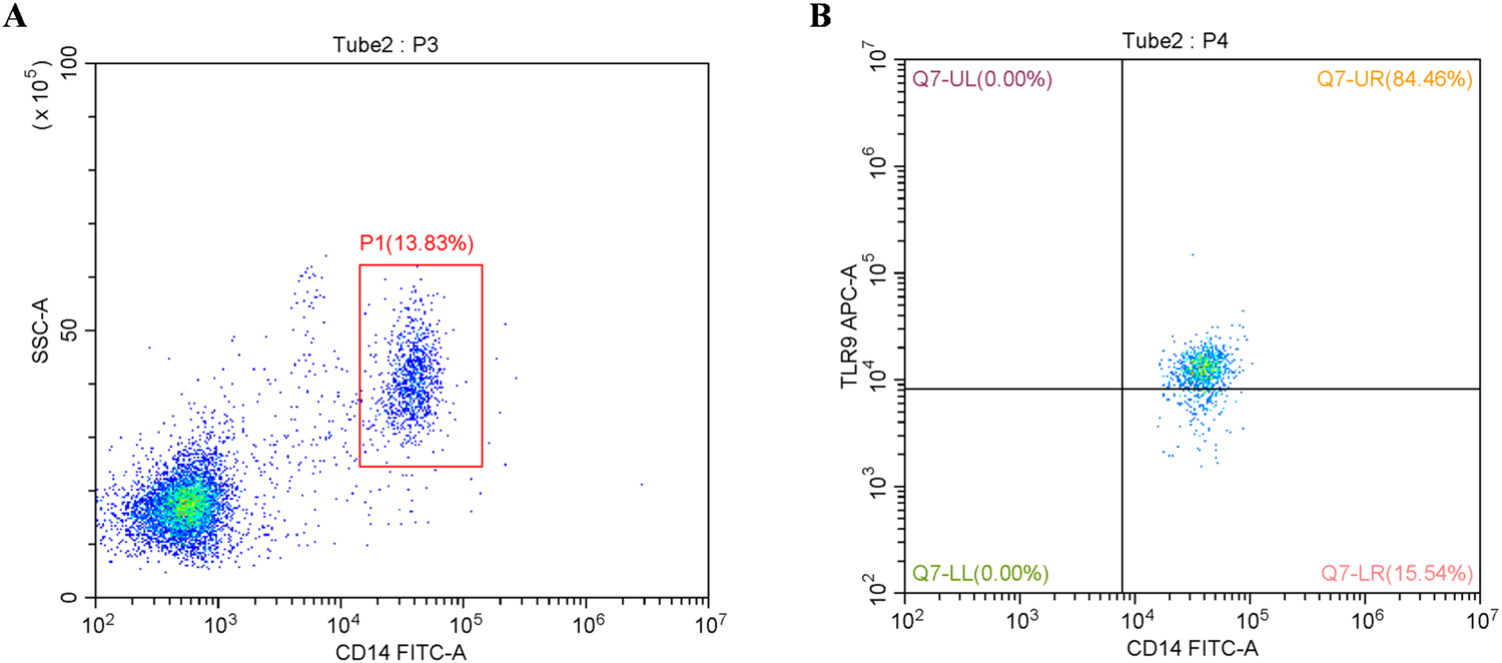
TLR9 is expressed in monocytes. PBMCs from healthy subjects were detected by flow cytometry to show the percentage of monocytes and TLR9 expression. (**A**) The percentage of monocytes in PBMCs was 13.83%. (**B**) TLR9 was positively expressed in 84.46% of monocytes.

A total of 200 subjects were enrolled and divided into the following four groups according to their status of HIV-1 infection and/or opiate abuse: Opiate+ HIV+ (n = 50), Opiate+ HIV− (n = 50), Opiate− HIV+ (n = 50), and healthy control (Opiate− HIV−, n = 50). Among the 200 subjects, 111 (55.5%) were male and 89 (44.5%) were female. Ages ranged from 18 to 60 years old (mean 31.2 ± 4.1 years). The subjects who were married or unmarried with cohabitation accounted for 44.5% of the total participants. There were no significant differences in age, gender, ethnicity, original residence, or marital status among the four groups (*P* > 0.05) (Table 2). The flow results show that TLR9 is expressed at a high level in monocytes (84.46% positive) (Fig. 1).

**Table 2:** Baseline of characteristics of four population groups.

Variable	Opiate+ HIV+ (n = 50) (%)	Opiate+ HIV−(n = 50) (%)	Opiate− HIV+ (n = 50) (%)	Control (n = 50) (%)	Total (n = 200) (%)	χ^2^ (F)	P
age (Mean, SD, years)	30.7 ± 4.5	31.5 ± 4.0	32.9 ± 4.2	29.8 ± 3.8	31.2 ± 4.1	2.67	0.095
gender						0.709	0.871
male	27 (54.0)	26 (52.0)	30 (60.0)	28 (56.0)	111 (55.5)		
female	23 (46.0)	24 (48.0)	20 (40.0)	22 (44.0)	89 (44.5)		
ethnicity						1.096	0.778
Han	39 (78.0)	36 (72.0)	37 (74.0)	40 (80.0)	152 (76.0)		
Others	11 (22.0)	14 (28.0)	13 (26.0)	10 (20.0)	48 (24.0)		
original residence (city)						13.853	0.128
Nanning	15 (30.0)	13 (26.0)	17 (34.0)	21 (42.0)	66 (33.0)		
Liuzhou	12 (24.0)	17 (34.0)	15 (30.0)	13 (26.0)	57 (28.5)		
Qinzhou	21 (42.0)	12 (24.0)	13 (26.0)	15 (30.0)	61 (30.5)		
Others	2 (4.0)	8 (16.0)	5 (10.0)	1 (2.0)	16 (8.0)		
marital status						7.511	0.057
unmarried	28 (56.0)	30 (60.0)	33 (66.0)	20 (40.0)	111 (55.5)		
married/cohabitating	22 (44.0)	20 (40.0)	17 (34.0)	30 (60.0)	89 (44.5)

### Opiate use was associated with higher viral load and lower CD4+ T cell count among HIV-1-infected subjects

**Figure 2:**
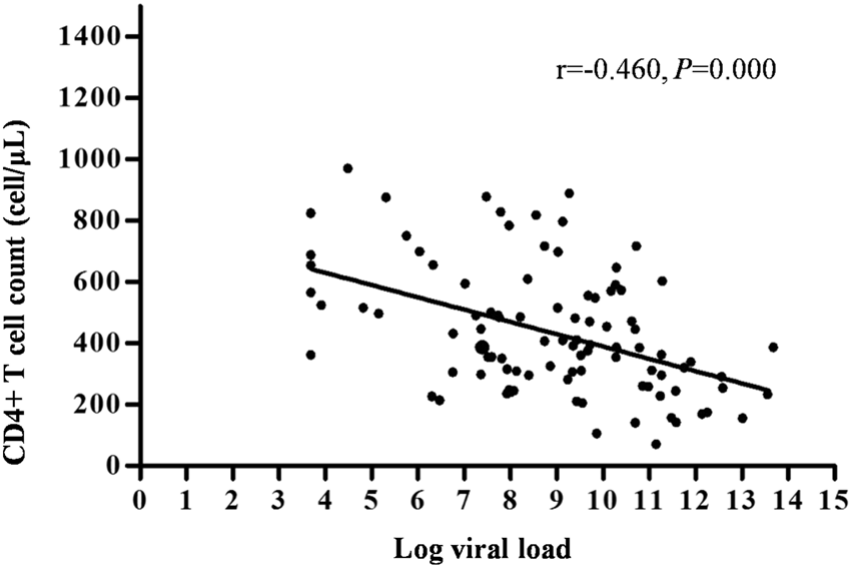
Scatter diagram for Log viral load and CD4+ T cell count. A Pearson’s correlation analysis was performed to analyze the relationship between CD4^+^ T cell count versus viral load (Log) among HIV-1-infected subjects. There was a negative correlation between Log viral load and CD4+ T cell count (r = −0.460, *P* = 0.000).

To evaluate the *in vivo* effects of opiate use on HIV-1 viral replication and cell immunity, we examined HIV-1 viral load and CD4+ T cell count among HIV-1-infected subjects. As indicated in Table 3, the mean viral load in the Opiate+ HIV+ group was (4.142 ± 0.988) × 10^3^ cps/mL, which was significantly higher than that in the Opiate− HIV+ group [(3.542 ± 0.978) × 10^3^ cps/mL] (*P* < 0.05). Conversely, the mean CD4+ T cell count in the Opiate+ HIV+ group was (4.036 ± 1.628) × 10^2^ cells/μL, which was significantly lower than that in the Opiate− HIV+ group [(5.031 ± 2.342) × 10^2^ cells/μL] (*P* < 0.05) (Table 3). Moreover, Pearson’s correlation analysis showed a negative correlation between the viral load and CD4+ T cell count (Fig. 2). These data indicated that opiate use was associated with a higher viral load and a lower CD4+ T cell count among HIV-1-infected subjects.

**Table 3:** HIV-1 viral load and CD4^+^ T cell count among HIV-1-infected subjects.

Variable	Opiate+ HIV+ (n = 50)	Opiate− HIV+ (n = 50)	t	*P*
Viral Load (Mean, SD, cps/mL)	(4.142 ± 0.988) × 10^3^	(3.542 ± 0.978) × 10^3^	2.991	0.004
CD4 Cell Count (Mean, SD, cells/μL)	(4.062 ± 1.628) × 10^2^	(5.031 ± 2.342) × 10^2^	2.106	0.036

### Opiate use and/or HIV-1 infection might inhibit TLR9 expression in PBMCs

**Figure 3:**
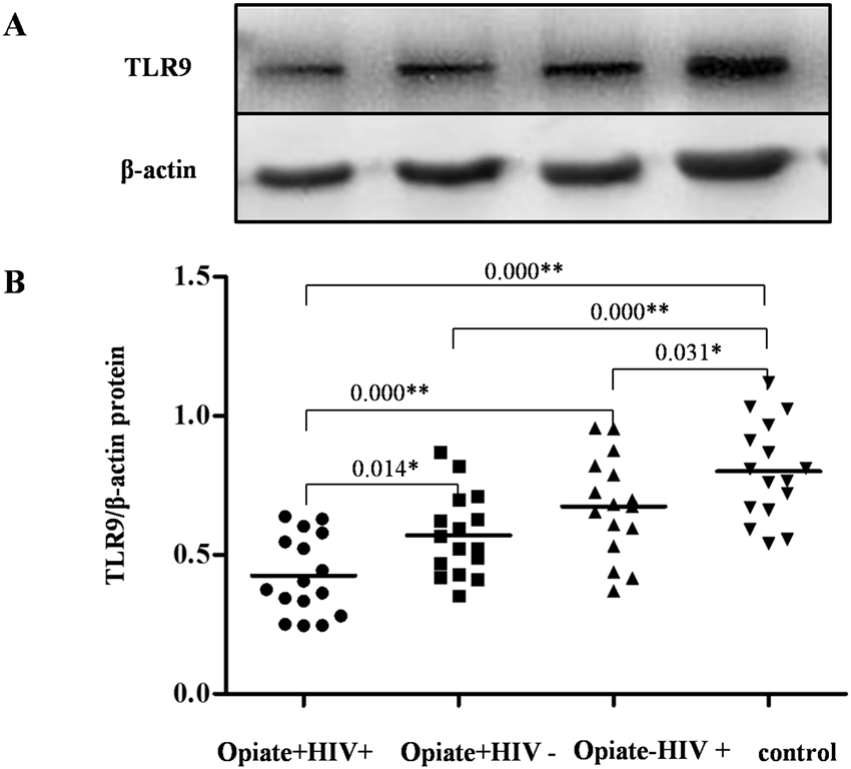
Effects of opiate use and/or HIV-1 infection on TLR9 expression in PBMCs. PBMCs were isolated from the 64 subjects, which were randomly selected from a total of 200 subjects, and were assigned to four groups: Opiate+ HIV+ (n = 16), Opiate− HIV+ (n = 16), Opiate+ HIV− (n = 16), and healthy controls (n = 16). Cellular proteins were prepared from PBMCs, and western blot was performed to examine the expression of TLR9 and β-actin. A representative western blot result for each of the four groups is shown. Densitometry analysis for TLR9 protein level was performed using ImageJ 1.44 software (National Institutes of Health). The data are expressed as the ratio of TLR9 protein level (gray value) to β-actin protein level (gray value). *P < 0.05, **P < 0.01.

To determine whether opiate use and/or HIV-1 infection had an impact on the TLR9 signaling pathway, we examined the expression of TLR9 protein in human PBMCs among the four groups by western blot. As shown in Fig. 3, the expression of TLR9 protein was highest in the control group compared with the other groups (*P* < 0.05). Among the HIV-1-infected subjects, the relative TLR9 protein level in the Opiate+ HIV+ group was significantly lower than that in the Opiate− HIV+ group (*P* < 0.05). Among opiate users, the relative TLR9 protein level in the Opiate+ HIV+ group was significantly lower than that in the Opiate+ HIV− group (*P* < 0.05). These data indicated that opiate use and/or HIV-1 infection inhibits TLR9 protein expression in PBMCs.

### Opiate use had no significant effect on the expression of MyD88 in PBMCs

**Figure 4:**
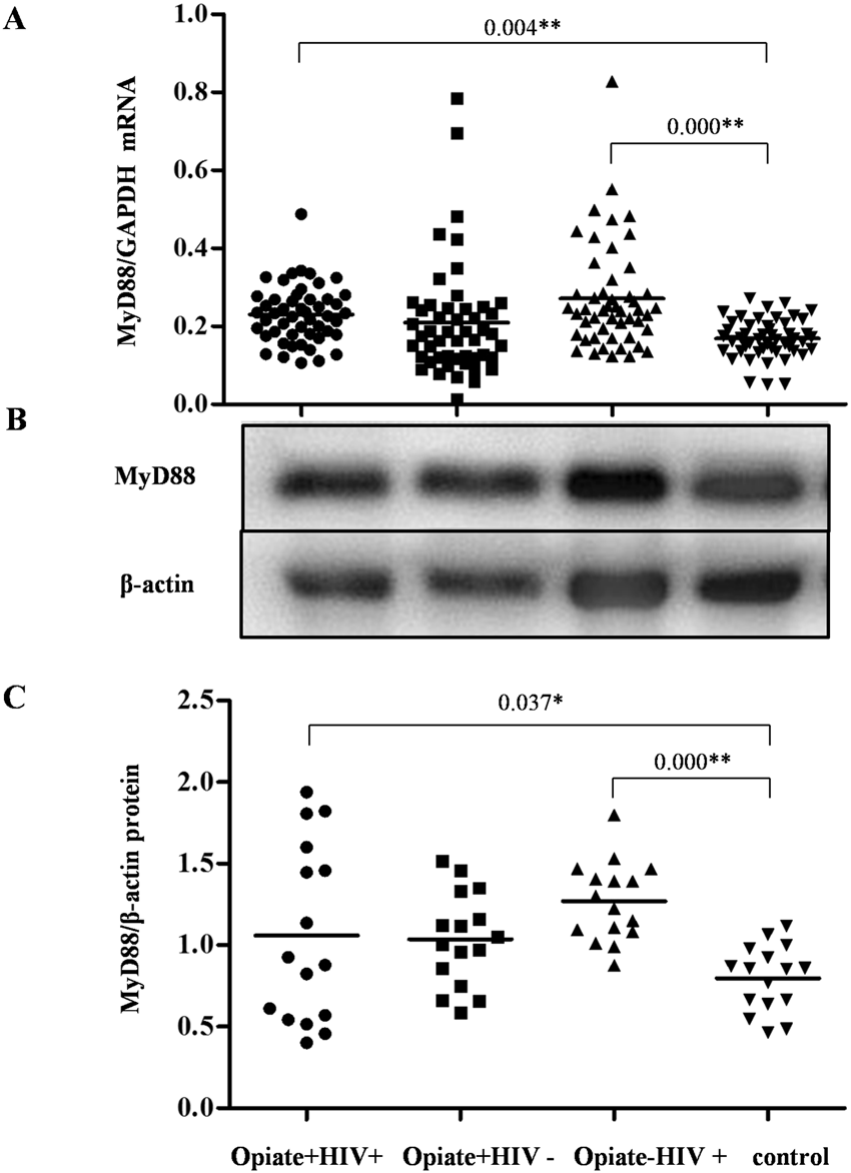
Effects of opiate use and/or HIV-1 infection on MyD88 expression in PBMCs. (**A**) The levels of MyD88 mRNA in PBMCs among four groups. PBMCs were isolated from the 200 subjects among four groups: Opiate+ HIV+ (n = 50), Opiate− HIV+ (n = 50), Opiate+ HIV− (n = 50), and healthy controls (n = 50). Total cellular RNA extracted from PBMCs was subjected to qRT-PCR to determine the mRNA levels of MyD88 and GAPDH. The data are expressed as the ratio of the MyD88 mRNA level to the GAPDH mRNA level. Mean values are indicated by horizontal bars, and significance was calculated by one-way ANOVA. (**B**) MyD88 protein expression in PBMCs among four groups. Sixteen samples for each group were randomly selected from 50 subjects in each group. Cellular proteins were prepared from PBMCs, and western blot was performed to examine the expression of MyD88 and β-actin. A representative western blot result for each of the four groups is shown. Densitometry analysis for the MyD88 protein level was performed using ImageJ 1.44 software (National Institutes of Health). The data are expressed as the ratio of MyD88 protein level (gray value) to β-actin protein level (gray value). *P < 0.05, **P < 0.01.

The expression of MyD88, one of key TLR9 adaptor proteins, was also detected. As shown in Fig. 4, there was no significant difference in the expression of MyD88 between the Opiate+ HIV+ group and the Opiate− HIV+ group or between the Opiate+ HIV− group and the control group (*P* > 0.05), indicating that opiate use had no significant effect on MyD88 expression. However, the expression of MyD88 in the Opiate+ HIV+ group and the Opiate− HIV+ group was significantly higher than that in the control group (*P* < 0.05, Fig. 4), indicating that HIV-1 infection promoted the expression of MyD88 in PBMCs.

### Opiate use disrupted IRF7 expression in PBMCs

**Figure 5:**
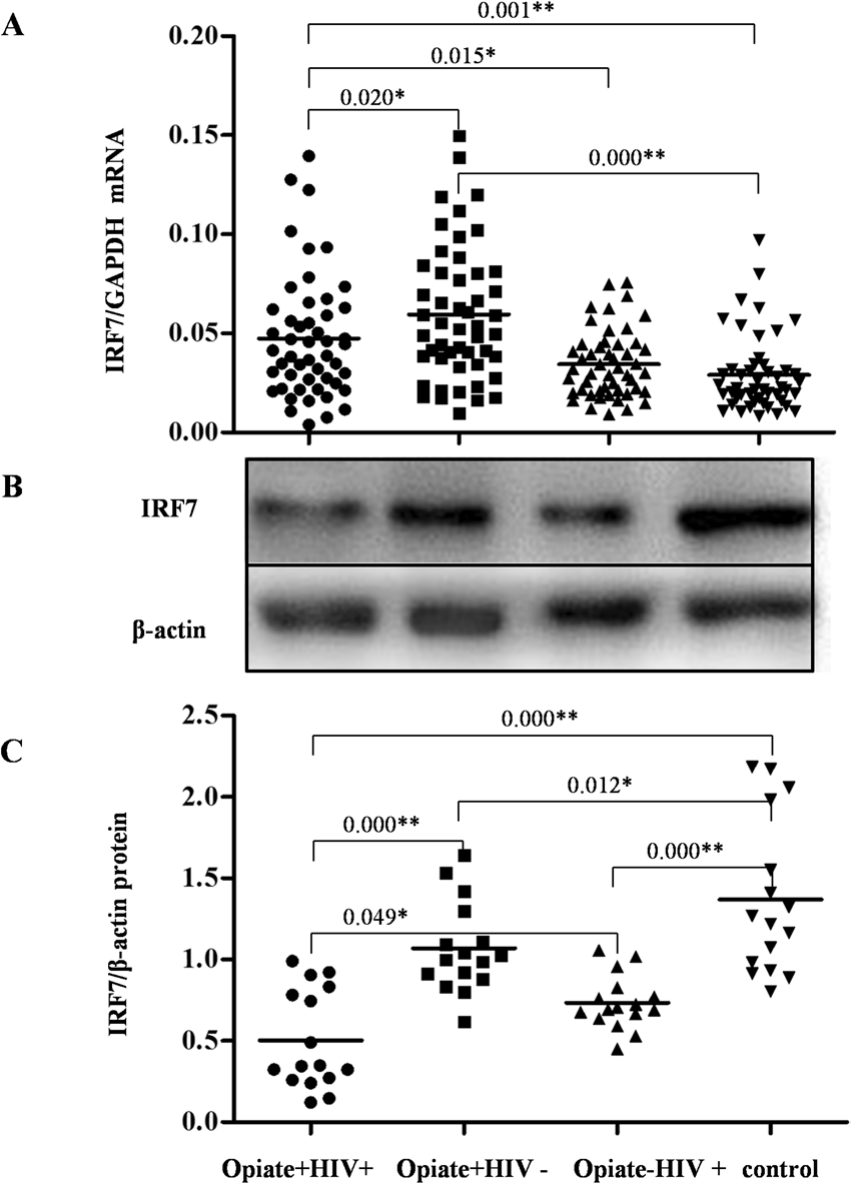
Effects of opiate use and/or HIV-1 infection on IRF7 expression in PBMCs. (**A**) The levels of IRF7 mRNA in PBMCs among four groups. PBMCs were isolated from the 200 subjects among four groups: Opiate+ HIV+ (n = 50), Opiate− HIV+ (n = 50), Opiate+ HIV− (n = 50), and healthy controls (n = 50). Total cellular RNA extracted from PBMCs was subjected to qRT-PCR to determine the mRNA levels of IRF7and GAPDH. The data are expressed as the ratio of the IRF7 mRNA level to the GAPDH mRNA level. Mean values are indicated by the horizontal bar, and significance was calculated by one-way ANOVA. (**B**) IRF7 protein expression in PBMCs among four groups. Sixteen samples for each group were randomly selected from 50 subjects in each group. Cellular proteins were prepared from PBMCs and western blot was performed to examine the expression of IRF7 and β-actin. A representative western blot result for each of the four groups is shown. Densitometry analysis for IRF7 protein level was performed using ImageJ 1.44 software (National Institutes of Health). The data are expressed as the ratio of the IRF7 protein level (gray value) to the β-actin protein level (gray value). *P < 0.05, **P < 0.01.

To further determine the potential impact of opiate use downstream of the TLR9 signaling pathway, we also examined the expression of IRF7, the key regulator that links the TLR9 signaling pathway and the type I interferon pathway. As shown in Fig. 5A, the levels of IRF7 mRNA in the Opiate+ HIV+ group and the Opiate+ HIV− group were higher than that in control group (*P* < 0.05), indicating that opiate use could induce an increase in IRF7 expression. In addition, among the HIV-1-infected subjects, the levels of IRF7 mRNA in the Opiate+ HIV+ group were markedly higher than that in the Opiate− HIV+ group (*P* < 0.05, Fig. 5A). Interestingly, western blot showed a contrary phenomenon in the expression of IRF7 protein. The results showed that either opiate use (Opiate+ HIV−) or HIV-1 infection (Opiate− HIV+) could lead to significantly decreased expression of IRF7 protein compared to the control group (*P* < 0.05, Fig. 5B). In addition, opiate use plus HIV-1 infection (Opiate+ HIV+) resulted in lower expression of IRF7 protein compared to the Opiate+ HIV− or Opiate− HIV+ group (*P* < 0.05, Fig. 5B).

### Opiate use was associated with lower levels of IFN-α in PBMCs and plasma

**Figure 6:**
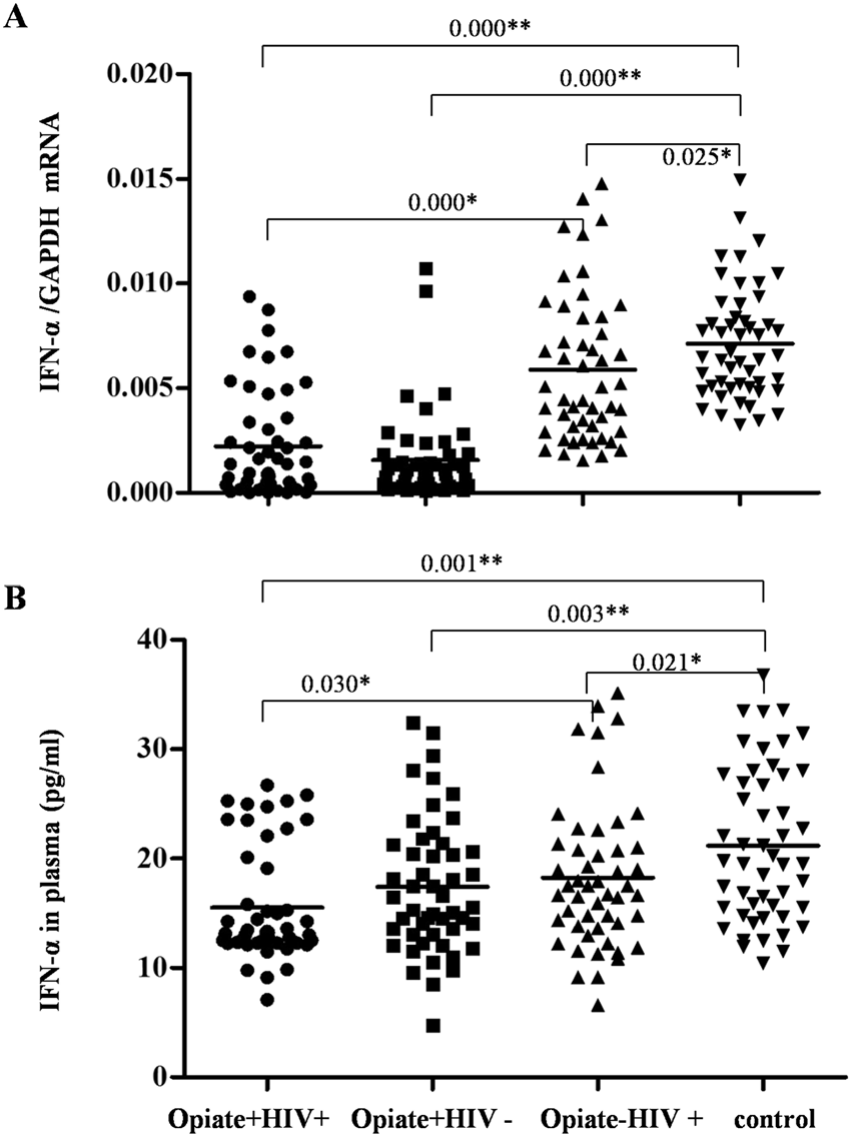
Effects of opiate use and/or HIV-1 infection on IFN-α expression in PBMCs and the levels of plasma IFN-α. (**A**) The levels of IFN-α mRNA in PBMCs among four groups. PBMCs were isolated from the 200 subjects among four groups: Opiate+ HIV+ (n = 50), Opiate− HIV+ (n = 50), Opiate+ HIV− (n = 50), and healthy controls (n = 50). Total cellular RNA extracted from PBMCs was subjected to qRT-PCR to determine the mRNA levels of IFN-α and GAPDH. The data are expressed as the ratio of the IFN-α mRNA level to the GAPDH mRNA level. Mean values are indicated by horizontal bars, and significance was calculated by one-way ANOVA. (**B**) The levels of IFN-α in plasma among four groups determined by ELISA. Plasma samples of all 200 subjects were submitted to ELISA to determine the protein levels of IFN-α. Mean values are indicated by horizontal bars, and significance was calculated by one-way ANOVA. *P < 0.05, **P < 0.01.

We further examined whether opiate use and/or HIV-1 infection affects the expression of IFN-α, an important type I interferon that is regulated by IRF7. As shown in Fig. 6, among the four groups, the levels of IFN-α mRNA in PBMCs and IFN-α protein in plasma were highest in the control group. The levels of IFN-α mRNA and IFN-α protein in the Opiate+ HIV+ group were lower than those in the Opiate− HIV+ group (*P* < 0.05), indicating that opiate use was associated with decreased levels of IFN-α in PBMCs and plasma. However, there was no significant difference between the Opiate+ HIV+ and Opiate+ HIV− groups (*P* > 0.05).

### Opiate use impaired ISG production in PBMCs

**Figure 7:**
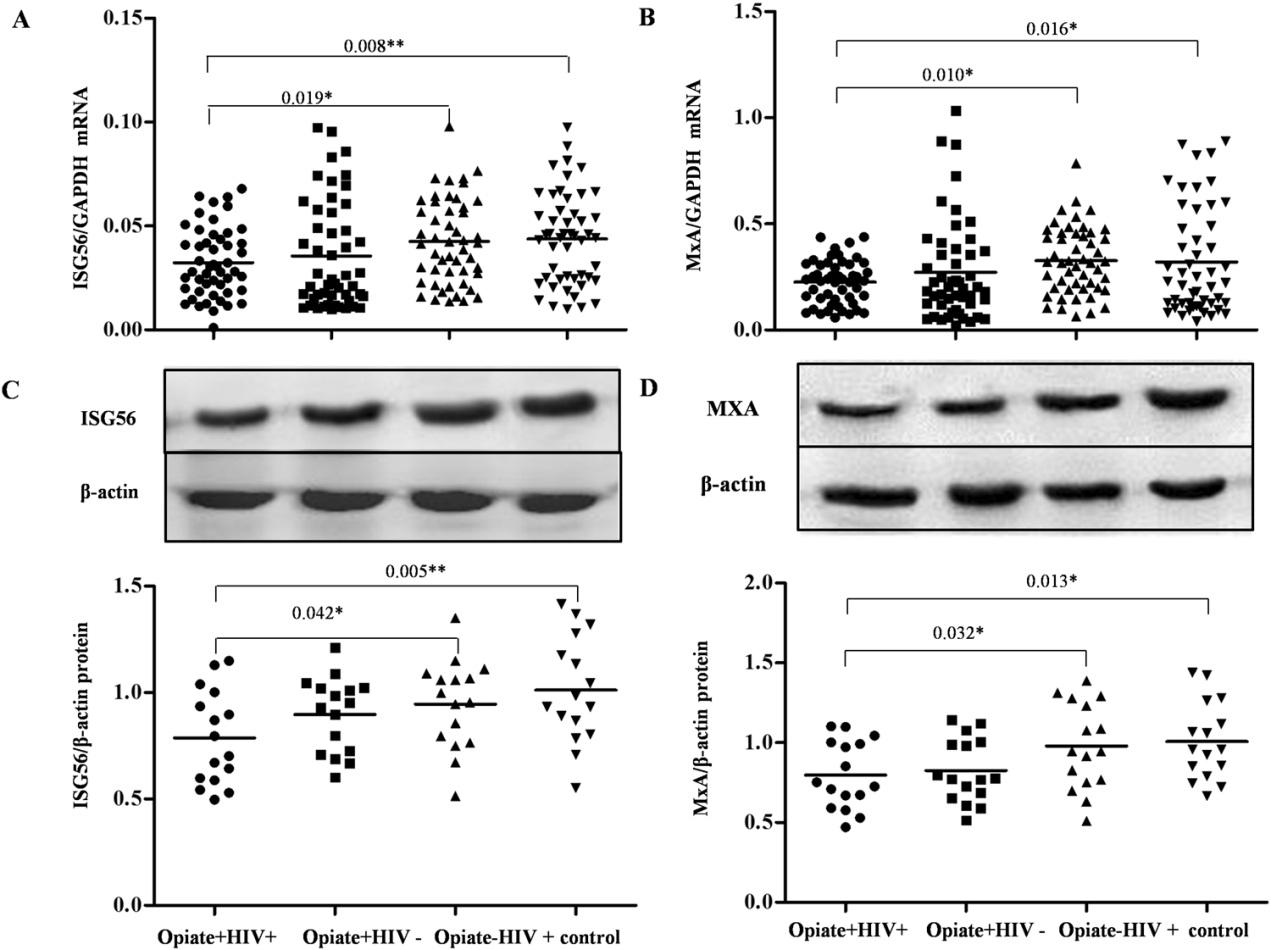
Effects of opiate use and/or HIV-1 infection on the expression of ISG56 and MxA in PBMCs. (**A**) and (**B**) The levels of ISG56 and MxA mRNA in PBMCs among four groups. PBMCs were isolated from the 200 subjects among four groups: Opiate+ HIV+ (n = 50), Opiate− HIV+ (n = 50), Opiate+ HIV− (n = 50), and healthy controls (n = 50). Total cellular RNA extracted from PBMCs was subjected to the qRT-PCR to determine the mRNA levels of ISG56 (**A**) and MxA (**B**). The data are expressed as the ratio of the ISG56 or MxA mRNA level to the GAPDH mRNA level. Mean values are indicated by horizontal bars and significance was calculated by one-way ANOVA. (**C**) and (**D**) The expression of ISG56 and MxA protein in PBMCs among four groups. Sixteen samples for each group were randomly selected from 50 subjects in each group. Cellular proteins were prepared from PBMCs, and western blot was performed to examine the expression of ISG56 (**C**) and MxA (**D**). A representative western blot result for each of the four groups is shown. Densitometry analysis for the protein expression of ISG56 and MxA was performed using ImageJ 1.44 software (National Institutes of Health). The data are expressed as the ratio of the ISG56 or MxA protein level (gray value) to the β-actin protein level (gray value). *P < 0.05, **P < 0.01.

Interferon stimulated genes (ISGs) are a class of antiviral cellular factors induced by type I interferons. Thus, we further investigated whether opiate use and/or HIV infection influences the expression of two ISGs, ISG56 and MxA. As shown in Fig. 7, opiate use significantly inhibited both ISG56 and MxA expression at the mRNA level (*P* < 0.05), whereas no difference in ISG56 and MxA mRNA levels was observed between the Opiate+ HIV+ and Opiate− HIV+ groups (*P* > 0.05, Fig. 7A,B). At the protein level, both opiate use and HIV-1 infection could significantly inhibit the expression of ISG56 and MxA (*P* < 0.05, Fig. 7C,D).

### Opiate use promoted HIV-1 replication in macrophages *in vitro* via inhibition of TLR9 signaling pathway

**Figure 8:**
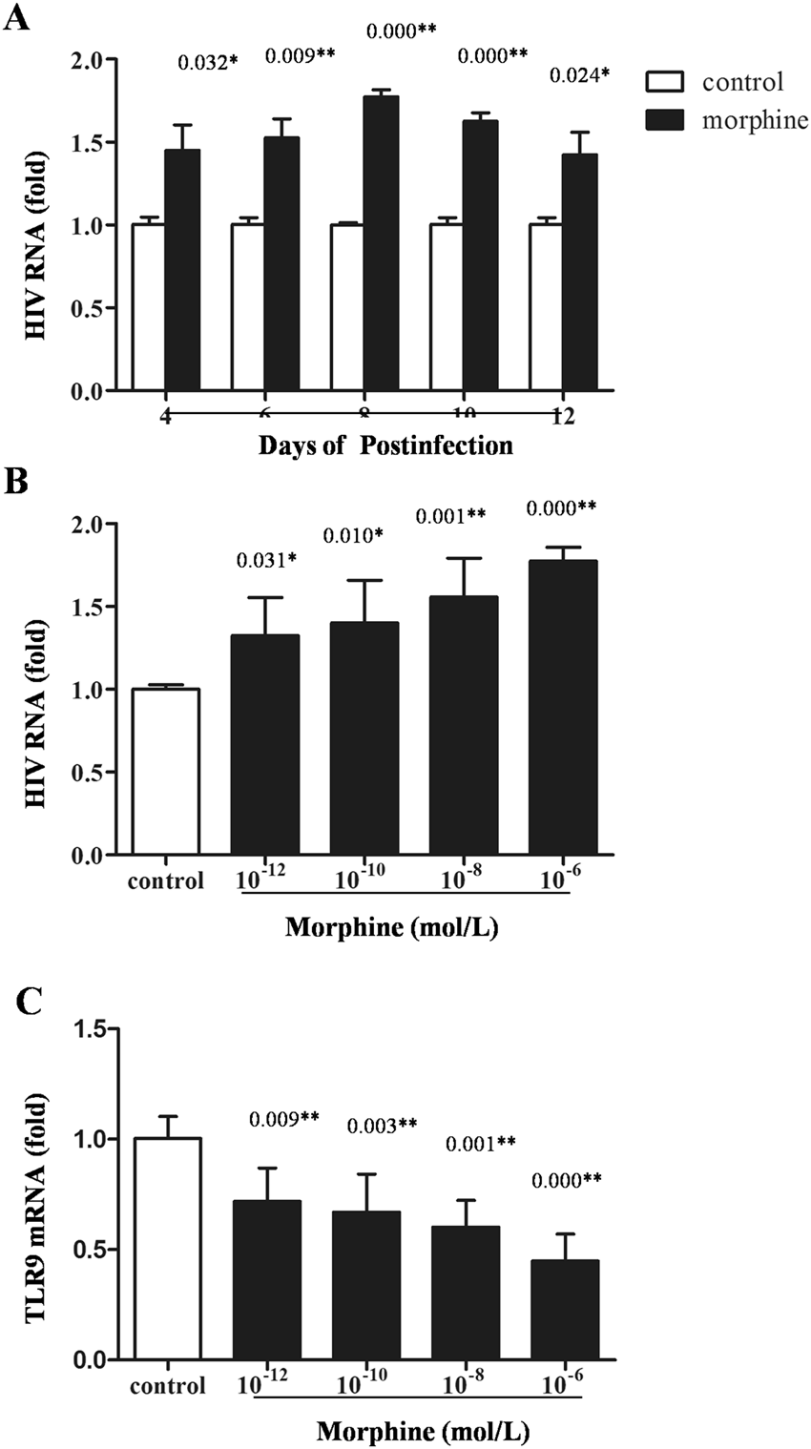
Effects of morphine on HIV-1 replication/TLR9 expression in macrophages. (**A**) Time-course effect of morphine on HIV-1 replication. Seven-day-cultured macrophages were incubated with or without 10^−6^ mol/L morphine for 24 hours before infection of the HIV Bal strain for 2 hours and then cultured for 12 days. The levels of HIV RNA in cultured supernatants were determined by qRT-PCR at the indicated time points after infection. (**B**) Dose-dependent effect of morphine on HIV-1 replication. Seven-day-cultured macrophages were incubated with or without morphine at indicated concentrations for 24 hours and then incubated with HIV Bal strain for 2 hours in the presence or absence of morphine. The levels of HIV RNA in cultured supernatants at day 8 postinfection. (**C**) Effect of morphine on HIV-1 replication. Seven-day-cultured macrophages were incubated with or without morphine at the indicated concentrations for 24 hours, and cultured cells were collected for TLR9 mRNA assay. In Fig. 8A,B,C, the data are expressed as fold of control (without morphine treatment, which is defined as 1). Mean values of three repeated experiments are indicated by horizontal bars, and significance was calculated by one-way ANOVA. *P < 0.05, **P < 0.01.

**Figure 9:**
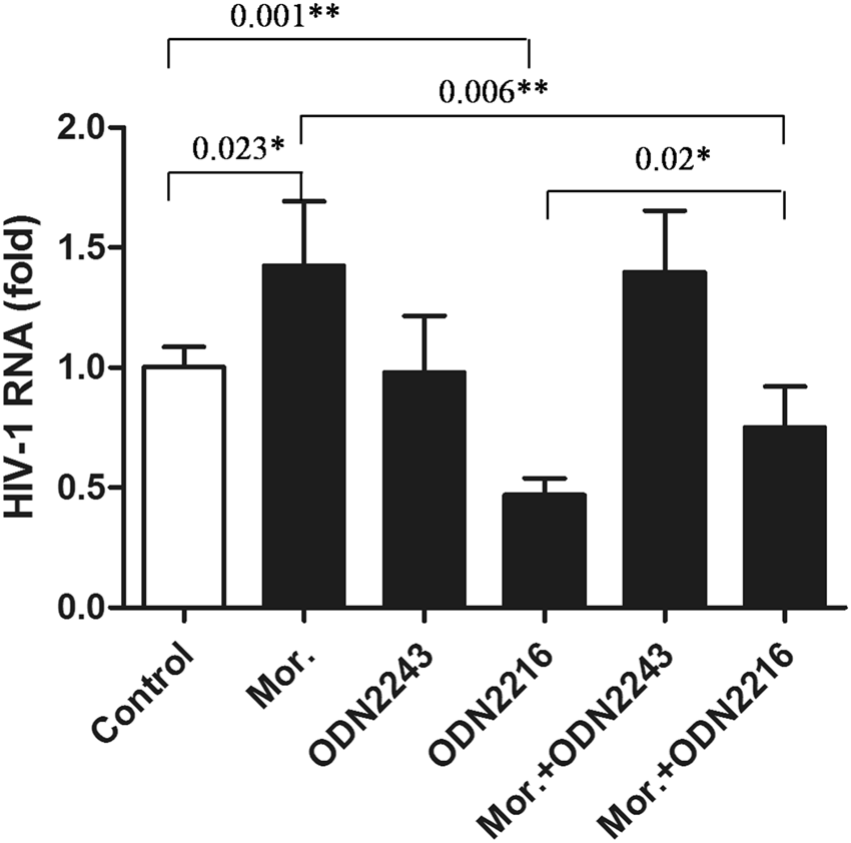
Morphine treatment promoted HIV-1 replication in macrophages *in vitro* via suppression of TLR9 signaling pathway. Seven-day-cultured macrophages were pretreated with ODN2216, the activator or inhibitor of the TLR9 signaling pathway, or its control, ODN2243, and then incubated with or without 10^−6^ mol/L morphine for 24 hours before infection of HIV Bal strain for 2 hours and then cultured for 12 days. The levels of HIV RNA in the cultured supernatants were determined by qRT-PCR. The data are expressed as fold of control (without morphine/ODN2216/ODN2243 treatment, which is defined as (**1**). Mean values of three repeated experiments are indicated by horizontal bars, and significance was calculated by one-way ANOVA. *P < 0.05, **P < 0.01.

We further detected the effects of morphine on HIV-1 replication in macrophages. As shown in Fig. 8A, morphine (10^−6^ mol/L) resulted in significant increases of HIV-1 replication in macrophages after different days postinfection in comparison to the control (*P* < 0.05). In addition, we found that HIV-1 replication significantly increased with the increase in morphine concentration after 8 days postinfection (*P* < 0.05), indicating a dose-dependent effect of morphine on HIV-1 replication in macrophages (Fig. 8B). Moreover, we observed that morphine treatment could lead to down-expression of TLR9 in macrophages in a dose-dependent fashion (*P* < 0.05, Fig. 8C). In addition, morphine treatment could compromise the ODN2216 (activator of the TLR9 signaling pathway)-induced anti-HIV effect (*P* < 0.05, Fig. 9), indicating that inhibition of the TLR9 signaling pathway by morphine plays a role in its enhancement of HIV-1 replication.

## Discussion

Chronic opiate use has been documented to compromise the immune system and thereby increase the risk of various infections, including HIV-1 infection^8–10^. Consistent with previous studies^7,39,40^, our results, which are based on a population study, showed that opiate use was associated with higher HIV-1 viral load and lower CD4^+^ T cell count among HIV-1-infected patients, providing compelling *in vivo* evidence to support the opinion that abuse of opiates acted as a cofactor in promoting HIV infection and disease progression. One of the possibilities for opiate action is that opiate can suppress the functional activity of innate immunity and consequently is detrimental to the host’s ability to eradicate pathogens^10^. Although sharing needles is a main of increased HIV-1 infectivity, the recruited subjects in this study had no needle sharing behaviors because of various programs in China, such as providing free needles and free needle exchange for addicts, which have existed for more than 10 years. Thus, the present study could rule out the effects of sharing needles in promoting HIV infection. In addition, lack of adherence to anti-retroviral therapy in drug abusers can also lead to increased viral loads. In this study, the drug abusers who had received anti-retroviral therapy were excluded. Thus, this study also excluded the possibility of lack of adherence to anti-retroviral therapy in promoting HIV infection. Moreover, our results showed that opiate use resulted in lower levels of IFN-α in PBMCs and plasma, which was in line with the findings of early studies that morphine treatment inhibited Sendai virus-induced IFN-α production in PBMCs^41^ and that morphine suppressed endogenous IFN-α expression in monocyte-derived macrophages and thus facilitated HIV-1 infection/replication^18^.

Accumulating evidence has confirmed that TLRs are key signal molecules for triggering an immune response against HIV-1 replication^42^. In our preliminary experiments, we found that morphine treatment did not result in significant changes in TLR3, 4, 7, and 8 expression (data not shown), except for TLR9; thus, we only examined the impact of opiate use on TLR9 expression in PBMCs to further determine the mechanisms involved in the enhancement of HIV replication by opiate use. The results showed that opiate use alone led to a significant reduction in TLR9 expression in PBMCs at either the mRNA level or protein level, which is consistent with previous findings in mice astroglia^43^. We also found that HIV-1 infection alone also led to the lower expression of TLR9 whether the HIV-1-infected subjects were opiate users or not, which is in agreement with the reports that TLR9 polymorphisms clinically affect HIV-1 progression^42,43^. Unsurprisingly, the expression of TLR9 in the Opiate+ HIV+ group was significant lower than that in the Opiate− HIV+ group in our study, implying that opiate use could enhance this inhibitory effect of HIV-1 infection on the expression of TLR9.

It is well known thatTLR9 can recognize the CpG motifs presented in bacterial or viral DNA^30,31^. When CpG or viral DNA binds to TLR9, TLR9 is stimulated and MyD88 and tumor necrosis factor (TNF) receptor-associated factor 6 (TRAF6) are recruited, leading to the MyD88-dependent activation of the transcription factor IRF7 and the subsequent transcriptional activation of type I interferon genes^44–46^. Beyond this, after viral infection, a subset of antiviral proteins, such as ISG56 and MxA, are induced in an interferon-dependent manner through the actions of IRF7 to respond directly to downstream signals of the TLR9 signaling pathway, thus restricting virus replication and modulating adaptive immunity. In addition, TLR9 recognizes microbial DNA components, and activation of this receptor following ligand binding induces the recruitment of MyD88 and the production of proinflammatory cytokines^47^. In this study, no differences in the expression of MyD88 in human PBMCs were observed. However, HIV-1 infection alone could induce MyD88 expression at both the mRNA and protein levels, which was in agreement with a previous study that HIV-1 Vpr induced MyD88-mediated IL-6 production and reactivated viral production from latency^48^. Furthermore, IRF7, which is constitutively expressed in pDCs, has a central role in type I interferon-mediated antiviral immunity. Our investigation showed that opiate use and HIV-1 infection up-regulated IRF7 expression at mRNA level; however, both of them could down-regulate IRF7 expression at the protein level. One of explanations for this phenomenon is that the effects of opiate use and HIV-1 infection on IRF7 expression are multifaceted. On the one hand, opiate use and HIV-1 infection could induce the expression of IRF7 mRNA. On the other hand, they might also inhibit IRF7 protein expression either at the IRF7 translation level or via post-translational modification or degradation. Interestingly, an obvious discrepancy in IRF7 mRNA and protein expression levels between the opiate+ and opiate− of the HIV-infected groups was observed in our study. The reasons for this phenomenon could due to the stages of HIV infection in the infected subjects. In the early period of HIV infection, immune cells can activate the TLR and type I interferon pathway with a normal function, whereas this pathway is suppressed when the host immune system is damaged by HIV infection in the late period of HIV infection. IRF7 is the key regulator of the type I-dependent immune pathway because it can induce IFN-α expression and the expression of many antiviral ISGs^49^. Thus, the decreased expression of IRF7 in PBMCs among opiate users provides a reasonable explanation for the inhibitory effect of opiate use on IFN-α expression as well as downstream ISG expression. Consistent with the results of the effects of opiate use on IRF7 and IFN-α expression, we found that opiate use inhibited the expression of two representative ISGs (ISG56 and MxA).

Lack of phenotypic characterization of PBMCs is a limitation of this study. To address this issue, experiments were carried out on monocytes/macrophages. Monocytes/macrophages are essential for the immune pathogenesis of HIV disease, acting as major target cells, reservoirs, vehicles to other tissues, and transmitters of the virus to CD4^+^ T cells, and thus, they participate in HIV infection during all stages of the disease^50^. Therefore, whether morphine enhances HIV infection of monocytes/macrophages merits further investigations. In our study, we first showed that TLR9 was highly expressed in human monocytes (Fig. 1) and that the TLR9 expressed in monocytes/macrophages is functional (Fig. 9); then, we exhibited that morphine treatment resulted in significant increases of HIV-1 replication in macrophages in a time- and dose-dependent manner. Activation of the TLR9 signaling pathway could effectively prevent morphine-enhanced HIV-1 replication *in vitro*. However, morphine treatment could down-regulate TLR9 expression in macrophages and compromise the anti-HIV effect of the activated TLR9 pathway (Figs 8 and 9). Taken together, these *in vitro* experiments further indicated that opiate use may inhibit the TLR9 signaling pathway, thus playing a key role in pathogenesis of HIV-1 infection.

In summary, based on a population study, our current findings reveal that opiate use may promote HIV-1 replication and may be a cofactor for the pathogenesis of HIV-1 infection. The data generated from our study provide a new understanding of the molecular mechanisms by which opiate use modulates immune function and enhances HIV-1 infection. In addition, HIV-1 infection itself may also alter the expression of key factors in the TLR9 signaling pathway and disrupt the TLR9 signaling pathway and subsequent innate responses, which provides a novel mechanism for HIV-1 immune escape. Although our epidemiological study suggests a possible association between opiate use and HIV-1 infection in the context of the TLR9 pathway, the molecular mechanisms remain to be further determined. Future animal studies are necessary to elucidate the molecular mechanisms involved in opiate use promoting HIV-1 infection through its impact on the TLR pathway.
